# Comparison of Hematopoietic Stem Cells Derived from Fresh and Cryopreserved Whole Cord Blood in the Generation of Humanized Mice

**DOI:** 10.1371/journal.pone.0046772

**Published:** 2012-10-11

**Authors:** Johanna Scholbach, Anett Schulz, Florian Westphal, Dietmar Egger, Anja Kathrin Wege, Ina Patties, Margarethe Köberle, Ulrich Sack, Franziska Lange

**Affiliations:** 1 Institute of Clinical Immunology, University of Leipzig, Leipzig, Germany; 2 Fraunhofer Institute for Cell Therapy and Immunology (IZI), Leipzig, Germany; 3 Vita34 AG, Leipzig, Germany; 4 Clinic of Gynecology and Obstetrics, Caritas Hospital St. Josef, University of Regensburg, Regensburg, Germany; 5 Department of Radiotherapy, University of Leipzig, Leipzig, Germany; Johannes Gutenberg University of Mainz, Germany

## Abstract

To study the function and maturation of the human hematopoietic and immune system without endangering individuals, translational human-like animal models are needed. We compare the efficiency of CD34^+^ stem cells isolated from cryopreserved cord blood from a blood bank (CCB) and fresh cord blood (FCB) in generating highly engrafted humanized mice in NOD-SCID IL2Rγ^null^ (NSG) rodents. Interestingly, the isolation of CD34^+^ cells from CCB results in a lower yield and purity compared to FCB. The purity of CD34^+^ isolation from CCB decreases with an increasing number of mononuclear cells that is not evident in FCB. Despite the lower yield and purity of CD34^+^ stem cell isolation from CCB compared to FCB, the overall reconstitution with human immune cells (CD45) and the differentiation of its subpopulations e.g., B cells, T cells or monocytes is comparable between both sources. In addition, independent of the cord blood origin, human B cells are able to produce high amounts of human IgM antibodies and human T cells are able to proliferate after stimulation with anti-CD3 antibodies. Nevertheless, T cells generated from FCB showed increased response to restimulation with anti-CD3. Our study reveals that the application of CCB samples for the engraftment of humanized mice does not result in less engraftment or a loss of differentiation and function of its subpopulations. Therefore, CCB is a reasonable alternative to FCB and allows the selection of specific genotypes (or any other criteria), which allows scientists to be independent from the daily changing birth rate.

## Introduction

Biomedical research for human diseases is often limited to *in vitro* research because of ethical reasons or, alternatively, uses different mouse models. To overcome the interspecies specific differences between mice and humans without endangering human beings, humanized mice offer a great opportunity to bridge this gap [1]. These mice generate a human immune system and are already successfully integrated in the study of human malignancies [2], [3], infectious diseases [4]–[6], or to study the human hematopoietic-lymphoid system [7]–[9]. Humanized mice are not only used for the investigation of disease pathogenesis, but also allow the testing of efficiency of new drugs or vaccines [10], [11].

The utility of humanized mice was enhanced by the humanization of immunodeficient mice lacking the (interleukin-2) receptor γ chain locus [7], [9], [12]. These mice develop no mature lymphocytes and NK cells [7] because the γ chain is an important component of many receptors for lymphoid-related cytokines and is crucial for the signaling through these receptors [13], [14]. The advantage of these mice is the lack of an adaptive immune system in addition to the lack of NK cells and an excellent engraftment of human cells [7], [15]. For the production of humanized mice, different human sources can be used e.g., hematopoietic stem cells from fresh cord blood (FCB) or more rarely, mobilized human stem cells (mSCs) [10]. In some cases humanized mice were generated by the combination of implantation of human tissue and transplantation of autologous stem cells in mice [4], [16]. One advantage to taking FCB instead of human tissue or mSCs is its accessibility and the higher amount of potential donors than tissue donors or donors for mSCs.

Nevertheless, it would be a big advantage to use cryopreserved cord blood (CCB) instead of FCB to humanize mice, because CCB allows the selection of cord blood with special features, like a defined genotype, e.g., for several disorders. Special HLA-II-molecules needed in Rheumatoid Arthritis [17], Multiple Sclerosis [18], [19] and Diabetes Type 1 [20], [21] or translocations on chromosomes required for the development of different types of leukemia [22] can be chosen in advance. Genotyping can be carried out before transplantation and helps to avoid the transplantation of unwanted cord blood samples. Furthermore, it makes studies independent of the necessity of daily donations of FCB. However, so far there are no studies, which compare the yield of CD34^+^ cells from CCB to the yield of CD34^+^ cells from FCB. Likewise, there are no published data about the function of the immune system in mice humanized with CD34^+^ cells from CCB in comparison with FCB. The present study closes this gap and shows that it is possible to separate CD34^+^ stem cells from CCB and to reconstitute a complete functional immune system in NOD-SCID IL2Rγ^null^ mice.

## Materials and Methods

### Ethic statements

Informed consent was obtained from all women, who donate the FCB and the study was approved by the local ethics committee (Ethics commission at the medical faculty of Leipzig; 121-11-18042011).

Animal experiments followed national guidelines for animal experiments and were approved by the local animal protection committee (Landesdirektion Leipzig; TVV 07/10).

### CD34^+^ cell separation from cryopreserved and fresh cord blood samples

Human cryopreserved cord blood was obtained from healthy full-term pregnancies with informed consent of the parents according to guidelines approved by the local ethics committee and according to the manufacturing authorization for cord blood transplants of Vita34 AG (Leipzig, Germany). The whole blood was cryopreserved in a computer controlled freezer (Ice-Cube, Sy-Lab, Austria) with the cryoprotectant DMSO (60%, Serumwerk Bernburg, Germany) in a final concentration of 5.5%. Fresh cord blood was obtained from healthy full-term pregnancies.

Cryopreserved human whole cord blood samples from Vita34 AG were defrosted in a 37°C water quench and stowed by warmed-up medium (RPM1640). After a centrifugation step (300 g, 5 min, 4°C), the supernatant was discarded and cells were washed in DNAse buffer (PBS with 2 mM MgCl_2_+10 µg/ml DNAse) to digest redundant DNA avoiding agglomeration of cells. After two additional washing steps, CD34^+^ stem cells were isolated by positive magnetic selection using the CD34-MicroBead-Kit, MS columns and a magnetic separator (Miltenyi Biotec GmbH, Bergisch Gladbach, Germany) according to the manufacturer's instructions.

Mononuclear cells (MNC) from FCB were obtained by Ficoll-Paque (PAN Biotech GmbH, Aidenbach, Germany) density gradient centrifugation. MNCs were washed three times with PBS containing 0.3 mM EDTA and CD34^+^ cells were isolated as described above.

### Generation of humanized mice

NOD.Cg-Prdc^scid^IL2rγ^tm1Wjl^/SzJ mice were obtained from Jackson Laboratory and were bred and kept under special pathogen free conditions at the University of Leipzig.

Newborn mice (24–48 h after birth) were irradiated with 1 Gy using a 200 kV X-ray machine (Gulmay) with a dose rate of 1.12 Gy/min and 3–5 hours later intrahepatically transplanted with 2–4×10^5^ CD34^+^ stem cells.

### Flow cytometry analysis

To validate the purity of the separated CD34^+^ cells, flow cytometry analyses were performed. Separated CD34^+^ cells were stained with FITC-conjugated anti-CD45 (clone 5B1) and PE-conjugated anti-CD34 antibodies (clone AC136) from Miltenyi Biotec (Bergisch Gladbach, Germany) for 30 min at 4°C. Afterwards cells were washed with PBS/2% FCS and fixed with 1% formaldehyde. Cells were analyzed on a FACSCanto II machine.

Six to eight weeks after transplantation of CD34^+^ cells, the blood of the mice was analyzed by flow cytometry to determine the reconstitution with human immune cells. Whole blood was stained with fluorescence-labeled antibodies for 30 min at 4°C. After lysis of the erythrocytes for 10 minutes at room temperature with BD lysis solution (BD Bioscience, Heidelberg, Germany), samples were washed two times with PBS/2% FCS and analyzed on a FACSCanto II machine. The cells were stained using the following antibodies purchased from BD Bioscience (Heidelberg, Germany): anti-CD3-FITC (clone UCHT1), anti-CD19-PE (clone HIB19), anti-CD8-PerCP (clone SK1), anti-CD16-PE-Cy7 (clone 3G8), anti-CD45-APC (clone HI30), anti-CD14-APC-Cy7 (clone MphiP9), anti-CD56-V450 (clone B159) and anti-CD4-Amcyan (clone SK3).

### Measurement of human immunoglobulins

Plasma samples from humanized mice were collected 19–21 weeks after transplantation of CD34^+^ cells from heart blood puncture and were stored at −80°C until measurement of human immunoglobulin concentrations. Levels of human IgM and human IgG antibody concentrations were analyzed in the plasma using cytometric bead array (BD Bioscience, Heidelberg, Germany) according to manufacturer's instructions.

### T cell proliferation assay

The spleens of the mice were removed and a single cell suspension was prepared by gently pressing the organs through a 70 µm cell strainer (BD Bioscience, Heidelberg, Germany). Spleen cells were washed once in PBS. To eliminate erythrocytes, spleen cells were lysed for two minutes in ACK lysis puffer (0.15 M NH_4_Cl, 1 mM KHCO_3_, 0.1 mM Na_2_-EDTA, pH 7.3). Lysis was stopped by addition of PBS and cells were washed twice in PBS. For monitoring the proliferation of the cells, spleen cells were labeled with CFDA-SE (Invitrogen, Darmstadt, Germany) as described previously [23]. Briefly, cells were incubated with CFDA-SE (2.5 µg/ml) for 10 min at 37°C and washed twice. The cells were placed into RPMI1640 containing stable glutamine, 100 U/ml penicillin, 100 µg/ml streptomycin and 10% heat-inactivated FCS (all PAA Laboratories GmbH, Cölbe, Germany) at a concentration of 2×10^6^ per ml and were incubated in flat-bottom 96-well tissue culture plates in a total volume of 200 µl. Cells were stimulated with plate-bound anti-CD3 (3 µg/ml) antibody (R&D, Wiesbaden-Nordenstadt, Germany). The cells were harvested from the tissue culture plate after 5 days of culture at 37°C and 5% CO_2_, stained with anti-CD3-PE (BD Bioscience, Heidelberg, Germany) and the proliferation of CD3 cells was analyzed by flow cytometry.

### Statistical analysis

Statistical analysis was done using GraphPad Prism 5.0 software (GraphPad Software, Inc., San Diego, USA). Differences in means or medians between groups were analyzed by Student's t-test or Mann-Whitney-U rank sum test where appropriate. Correlations were evaluated using the Pearson product moment correlation or the Spearman rank correlation coefficient method.

## Results

### CD34^+^ cells can be obtained from blood bank CCB, but show a higher variation in purity dependent on the total MNC number

**Figure 1:**
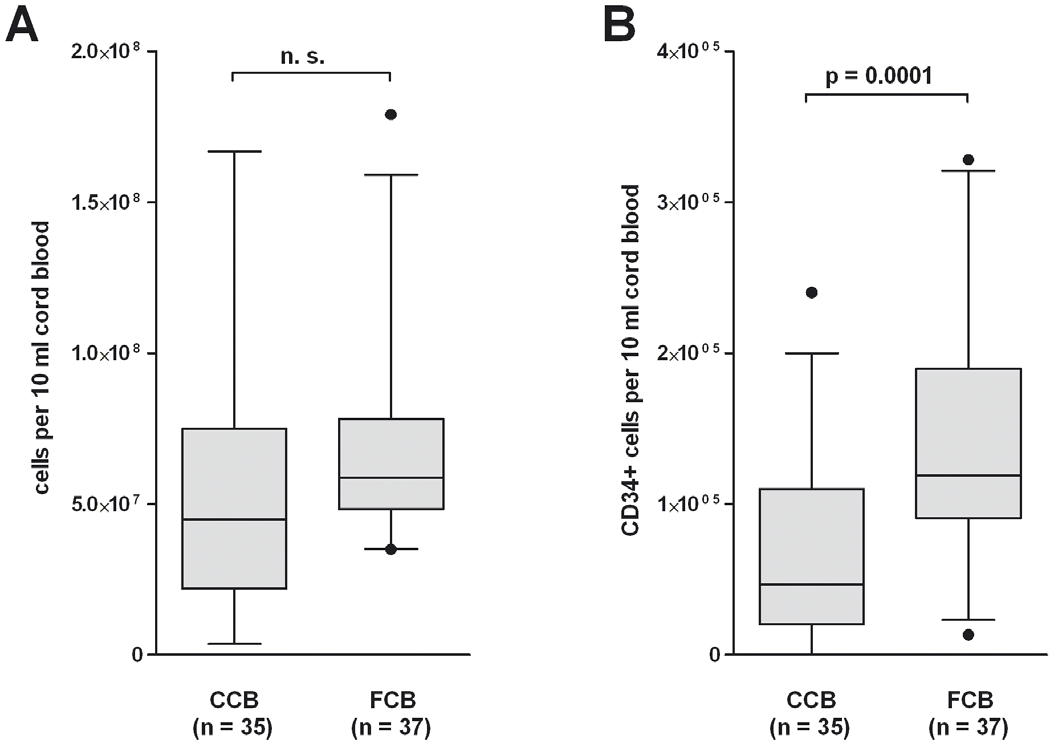
Differences and parallelism in the yield of CD34^+^ -cell-purifications from FCB and blood bank CCB. (A) MNC were isolated from CCB or FCB by washing with DNAse buffer or Ficoll-paque density gradient centrifugation as described in materials and methods. The total number of MNC per 10 ml cord blood is shown. (B) CD34^+^ cells were isolated from MNC by a positive magnetic separation of CD34^+^ cells. The number of CD34^+^ cells per 10 ml cord blood is shown.

**Figure 2:**
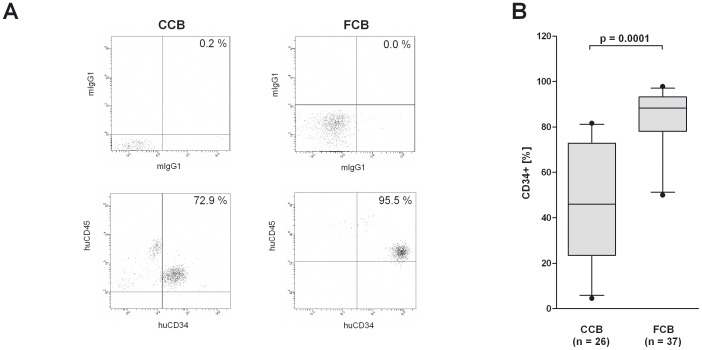
Differences and parallelism in the purity of CD34^+^ -cell-purifications from FCB and blood bank CCB. (A) MNC were isolated from FCB and blood bank CCB by washing with DNAse buffer or Ficoll-paque density gradient centrifugation as described in materials and methods. CD34^+^ stem cells were isolated from MNC by a positive magnetic separation of CD34+ cells and the purity was analyzed by flow cytometry. Dot plots depict the percentage of CD34^+^ cells of one representative separation from CCB and FCB (lower panels). The quadrant was set according to the isotype controls (upper panels). (B) The bar chart depicts the percentage of CD34^+^ cells after isolation of CD34^+^ cells from 26 CCB and 37 FCB samples. Box plots depict median and 5–95% percentile. Level of significance is given. n. s. = no significance.

**Figure 3:**
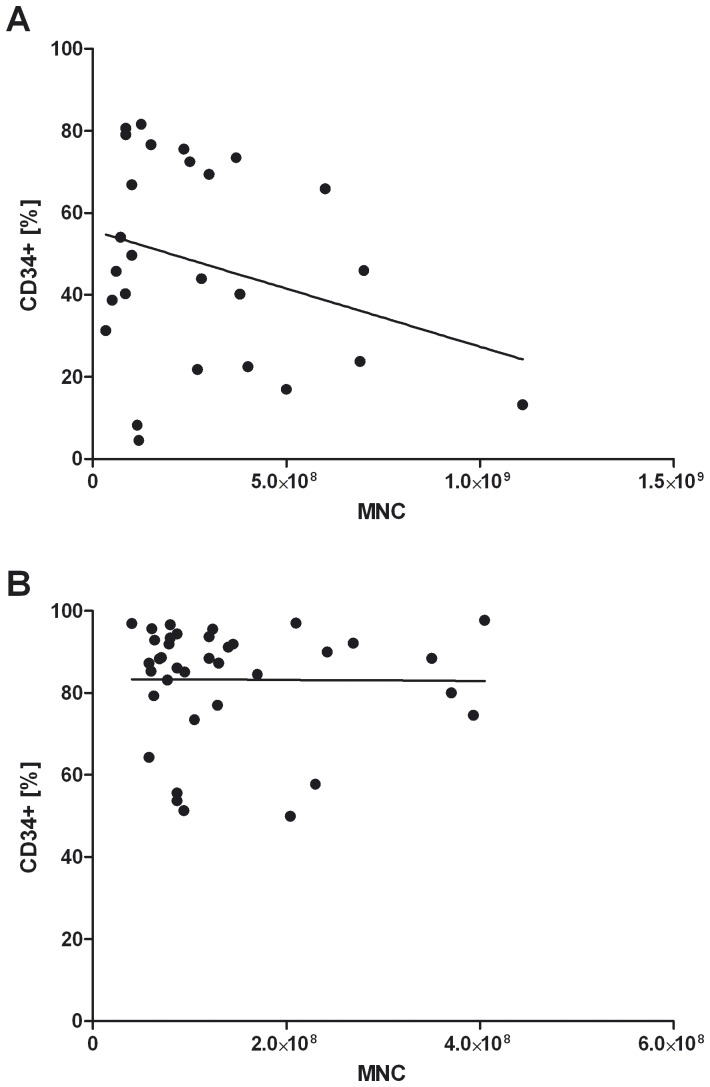
The purity of CD34^+^ separation decreases with an increasing number of MNC in CCB. MNC were isolated from CCB or FCB by washing with DNAse buffer or Ficoll-paque density gradient centrifugation as described in materials and methods. CD34^+^ stem cells were isolated from MNC by a positive magnetic separation of CD34^+^ cells and the purity was determined by flow cytometry. The correlations between the number of MNC and the purity of the CD34^+^ separation for CCB (A; no significance) and FCB (B) are shown. Correlations were evaluated using GraphPad Prism software and the Spearman rank correlation coefficient method.

Isolation of CD34^+^ cells from cord blood has already been published several times [24]–[26]. Due to the fact that the use of CCB allows a previous selection of the cord blood by any criteria in addition to the independency of daily donation, we tested the feasibility of blood bank CCB for CD34^+^ isolation. From 35 different CCB and 37 FCB samples, mononuclear cells were extracted as described in materials and methods. The average amount of extracted mononuclear cells was comparable between CCB and FCB (5.6×E7 vs. 6.7×E7; Figure 1A). Concerning the amount of CD34^+^ cells, the rate was lower in CCB than in FCB (6.5×E4 vs. 1.4×E5; p = 0.0001; Figure 1B). From 26 CCB and 37 FCB samples, the purity of isolated CD34^+^ cells was determined. Due to the amount of sample material, the purity of isolated CD34^+^ cells could not be examined for all CCB samples. In CCB, about 47.8% of the isolated cells were verified to be CD34^+^ (Figure 2A, 2B). In the control group with FCB, the purity of the isolated CD34^+^ cells was higher (83.3% CD34^+^ cells; p = 0.0001; Figure 2A, 2B). As might be expected with an increasing count of mononuclear cells, the total yield of CD34^+^ cells increased (data not shown). In contrast, a negative trend between the total amount of mononuclear cells and the purity of CD34^+^ cells was evident in CCB (not significant; Figure 3A) but not in FCB samples (Figure 3B).

### Reconstitution of a human immune system in NOD-SCID IL2Rγ^null^ mice after intrahepatic injection of CD34^+^ cells from blood bank CCB

**Figure 4:**
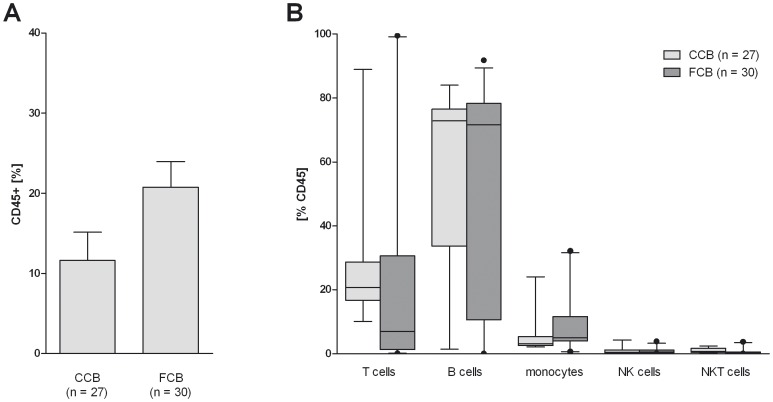
Comparison of reconstitution level in humanized mice transplanted with either blood bank CCB or FCB. The reconstitution of NOD-SCID IL2Rγ^null^ mice with human immune cells were analyzed 6–8 weeks after transplantation of CD34^+^ cells by flow cytometry analysis of the peripheral blood. (A) The bar charts depict the percentage of huCD45^+^ cells in the live gate and (B) the percentage of different cell populations among huCD45^+^ cells. Bar plots depict the mean + SEM (A) or median and 5–95% percentile (B).

To generate humanized mice, CD34^+^ cells were isolated from CCB and FCB and injected into the liver of newborn irradiated NOD-SCID IL2Rγ^null^ (NSG) mice. After six to eight weeks, the reconstitution level was determined by flow cytometry analysis of peripheral blood samples. The intrahepatic transplantation of CD34^+^ stem cells from CCB into NOD-SCID IL2Rγ^null^ mice resulted in the successful reconstitution of a human immune system in these mice. Humanized mice showed about 11% human CD45^+^ cells in the peripheral blood (Figure 4A). The rate of reconstitution of a human immune system in mice, which were humanized with CD34^+^ cells from FCB was slightly higher (20.8% human CD45^+^ cells in the peripheral blood). Furthermore, the maturation of the different subsets of immune cells, including B cells, T cells and monocytes in both groups, was determined by flow cytometry (Figure 4B). Mice from both groups showed individual differences in immune cell distribution. Likewise, mice aged up to two months developed B cells in a higher percentage than T cells independent of the cord blood source (Figure 4B).

### Functional human B and T cells maturate in blood bank CCB transplanted humanized NOD-SCID IL2Rγ^null^ mice

**Figure 5:**
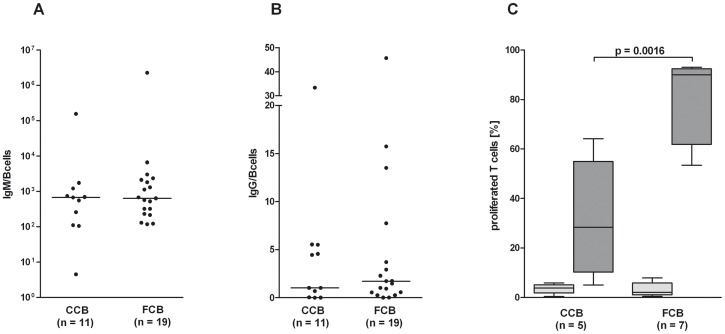
Humanized mice generated functional human B and T cells. (A, B) The concentration of human IgM and IgG in the plasma of humanized mice was determined by cytometric bead array 19–21 weeks after intrahepatic transplantation of CD34^+^ cells from CCB or FCB. (C) Spleen cells were labeled with CFDA-SE and cultured for 5 d in the presence (dark gray bars) or absence (gray bars) of an anti-CD3 antibody. Proliferation of cells was determined by flow cytometry. Box plots depict median and 5–95% percentile. Level of significance is given.

To investigate whether the reconstituted human immune cells are functional, spleen cells were separated and stimulated with anti-CD3 antibodies to analyze the T cell functionality. As shown in Figure 5C, T cells responded to the stimulation with proliferation. Interestingly, T cells from mice, which were humanized with CD34^+^ cells from FCB, showed a significantly higher proliferation rate compared to T cells from mice, which were humanized with CD34^+^ cells from CCB (p = 0.0016; Figure 5C).

To determine the function of the human B cells, plasma from these humanized mice was analyzed for human IgM and IgG antibody production by cytometric bead array. As expected, the concentration of IgM (Figure 5A) was significantly higher compared to the level of IgG (Figure 5B). These results were independent of whether FCB or CCB was used for the transplantation (Figure 5A, 5B). However, the concentration of antibodies in the plasma demonstrated a strong individual variation.

## Discussion

Translational animal models are helpful to improve the knowledge of the human hematopoietic/immune system while avoiding interventions on human patients. One possibility to generate mice with a human immune system is the intrahepatic injection of CD34^+^ stem cells, isolated from FCB [2], [27], [28]. The aim of the present study was to analyze the separation of CD34^+^ stem cells from blood bank CCB and its reconstitution ability in NSG mice. The use of this blood source allows a previous selection on genotype or any criteria and also provides a source that is more independent of donor frequency.

The present study shows that it is possible to isolate CD34^+^ stem cells from blood bank CCB for the generation of functional humanized mice. We were able to prove that there is no significant loss in the amount of mononuclear cells in CCB compared to FCB (Figure 1A). Nonetheless, after purification, the amount and the purity of CD34^+^ stem cells are lower in blood bank CCB compared to FCB (Figure 1B, 2A, 2B). This might be due to the fact that the proportion of DMSO as freezing protection and cord blood is unfavorable in blood bank CCB. This may result in an increased number of destroyed cells and thus in an increased release of DNA during thawing. Even if DNAse buffer is used, the remaining DNA agglutinates the living cells, which results in a lower yield and purity of the extracted CD34^+^ stem cells. In addition, it is possible that the unfavorable proportion of DMSO and cord blood causes an increased death of CD34^+^ stem cells, which results in the lower yield. Our data show that there is a negative trend between the amount of mononuclear cells used and the purity of the extracted CD34^+^ cells in blood bank CCB (Figure 3A). In FCB, the amount of mononuclear cells does not affect the purity (Figure 3B) and therefore seems to be the better source for CD34^+^ stem cells if there are no special requirements on the genotype of the donor.

Despite the lower yield and purity of CD34^+^ stem cells from CCB, the present study was able to prove that humanized mice with a functional human immune system can be generated by transplanting CD34^+^ stem cells from CCB (Figure 4). The reconstitution level between CCB and FCB is comparable and does not reveal differences in the distribution of the different subpopulation (B cells, T cells, monocytes, NK cells and NKT cells; Figure 4B).

The composition of the immune cell populations is as expected from literature, especially considering the high amount of B cells compared to the other cell populations like T cells and NK cells [27], [29]. The low amount of T cells might be due to the early time point that the blood samples were analyzed. Choi et al. describe that B cells gradually decrease whereas T cells increase in a time-dependent manner after transplantation of CD34^+^ stem cells [27].

Functionality of the reconstituted human immune cells was demonstrated by measuring the ability of B cells to produce IgM and IgG antibodies and by analyzing the ability of T cells to proliferate after stimulation. The B cells of both groups are able to produce high amounts of human IgM antibodies (Figure 5A) but only a low amount of human IgG antibodies (Figure 5B). This leads to the conclusion that the B cells are immature, which is in accord with the animal husbandry in a specific pathogen free facility and the already published literature [9], [30], [31]. In general, T cells from humanized mice are functional since they are able to proliferate after stimulation with superantigens or polyclonal mitogens [32], [33]. The present study confirms the functionality of T cells by their ability to proliferate in response to anti-CD3 antibody. Interestingly, there is a remarkable difference between T cells of mice humanized with CCB to those transplanted with FCB. T cells from mice humanized with CD34^+^ stem cells from FCB demonstrated a higher proliferation rate compared to mice humanized with CCB. This might be due to the negative impact of the freezing procedure on the stem cells, which in turn requires more time for the generation of fully functional human T cells in the mice environment.

In conclusion, it is possible to isolate CD34^+^ stem cells from CCB and the intrahepatic transplantation of these cells results in a successful humanization of NOD-SCID IL2Rγ^null^ mice. Therefore this study has proven the feasibility to use blood bank CCB for scientific questions, which need to test the genotype in advance and might offer new opportunities for researchers using the translational model of humanized mice in the future, independently from the frequency of daily birth rates.
